# Long term visuo-vestibular mismatch in freely behaving mice differentially affects gaze stabilizing reflexes

**DOI:** 10.1038/s41598-020-77026-w

**Published:** 2020-11-18

**Authors:** Filipa França de Barros, Louise Schenberg, Michele Tagliabue, Mathieu Beraneck

**Affiliations:** Integrative Neuroscience and Cognition Center, CNRS, Université de Paris, 75006 Paris, France

**Keywords:** Long-term memory, Brainstem, Cerebellum, Vestibuloocular reflex, Learning and memory, Motor control, Oculomotor system, Neuroscience, Sensorimotor processing, Reflexes

## Abstract

The vestibulo-ocular reflex (VOR) and the optokinetic reflex (OKR) work synergistically to stabilize gaze in response to head movements. We previously demonstrated that a 14-day visuo-vestibular mismatch (VVM) protocol applied in freely behaving mice decreased the VOR gain. Here, we show for the first time that the OKR gain is also reduced and report on the recovery dynamics of both VOR and OKR after the end of the VVM protocol. Using sinusoidally-modulated stimulations, the decreases in VOR and OKR were found to be frequency-selective with larger reductions for frequencies < 0.5 Hz. Constant-velocity OKR stimulation tests demonstrated that the persistent components of the OKR were not modified while the transient, initial responses were. To identify the signals driving VOR and OKR reductions, we compared the responses of mice exposed to a high-contrast and no-contrast VVM. Despite being more robust in the high-contrast conditions, reductions were largely comparable and recovered with a similar time course. An analysis that directly compared VOR and OKR responses revealed that, alterations in the VOR were of significantly larger amplitude with significantly slower dynamics of recovery. Our findings are evidence for a frequency-selective influence of visual signals in the tuning of gaze stabilizing reflexes in normal mice.

## Introduction

During everyday life, natural head movements in mammals cover a large range of frequencies and velocities^1–3^. To avoid blurry vision, image displacements on the retina are minimized by compensatory eye movements. These eye-in-space movements are referred to as gaze stabilization eye movements, which result from the transformation of sensory signals into extraocular motor commands^4^. Vertebrates possess two gaze stabilizing reflexes -the optokinetic reflex (OKR) and the vestibulo-ocular reflex (VOR)—that act synergistically to compensate for environmental and self-movements. The OKR responses rely on direction-selective retinal ganglion cells that are efficient for relatively slow motions of the visual scene (± 3º/s in mice)^5,6^. Consequently, the OKR gain is inversely proportional to the velocity of the visual stimulus^7^. On the other hand, the vestibular acceleration-sensitive neurons responsible for VOR are more sensitive to mid-to-high frequency range head motions^8^. In addition, the OKR can respond to constant-velocity visual motions while the vestibular system encodes only non-constant, transient head velocities^6^. The optokinetic and vestibulo-ocular reflexes are therefore functionally complementary, their combination enables efficient gaze stabilization and allows to discriminate self-generated from externally imposed movements in most naturally encountered situations.

The VOR works as an open-loop system^9,10^: it is completely functional in the dark, i.e., inner ear vestibular signals generate compensatory eye movements even in the absence of visual feedback. In rodents, the initial development of the VOR relies on the early maturation of the vestibular circuitry even before eye-opening (P12-14)^11–14^. Nevertheless, visual inputs are critical for the development and proper functioning of VOR: its fine-tuning depends on the visual feedback that informs on the efficacy of the compensatory eye movements. In the absence of vision, such as in congenitally or adventitiously blind people, the VOR is impaired^15^. The gain of the vestibulo-ocular reflex improves after the opening of the eyes in mice, while the phase shifts toward smaller phase leads^9^. In addition, vision critically influences the time constant of the velocity storage^16^, the development of vestibular nuclei neurons^17^ and the acquisition of their plastic properties^14,18,19^.

We recently reported a visuo-vestibular mismatch (VVM) protocol which consists in a high-contrast patterned device wore by the animal for 14 consecutive days while freely behaving in its cage^20^. Using this VVM, we demonstrated in adult mice that the long-term visual perturbation leads to neural modifications in the direct vestibulo-ocular pathway^21^. Specifically, VVM led to a drastic reduction of the VOR, associated with changes in the efficacy at the synapse between vestibular afferents and medial vestibular neurons as well as modifications of the intrinsic properties of a subpopulation of central vestibular neurons^21^.

Nevertheless, our previous study let several questions unanswered. First, it remains unclear which neural signals (*e.g*. oculomotor error/retinal slip)^22–24^ drive VVM plasticity. To answer to this question in the present study mice were tested either with a high- or no-contrast VVM device. If the retinal slip is among the signals driving plasticity, we expect different VOR gain reduction in the 2 conditions. Second, whether the changes in the VOR are paralleled with changes in the OKR remains elusive. To better understand the interplay between VOR and OKR, both reflexes were measured throughout the protocol. Lastly, few studies have explored the capacity of the VOR to recover following alteration in mice^25^. To determine how VOR and/or OKR recover after VVM, the gaze stabilizing reflexes were measured before the VVM protocol, right after its conclusion, and until recovery to pre-VVM levels.

## Results

### Effect of the visuo-vestibular mismatch and recovery of the vestibulo-ocular reflex

**Figure 1:**
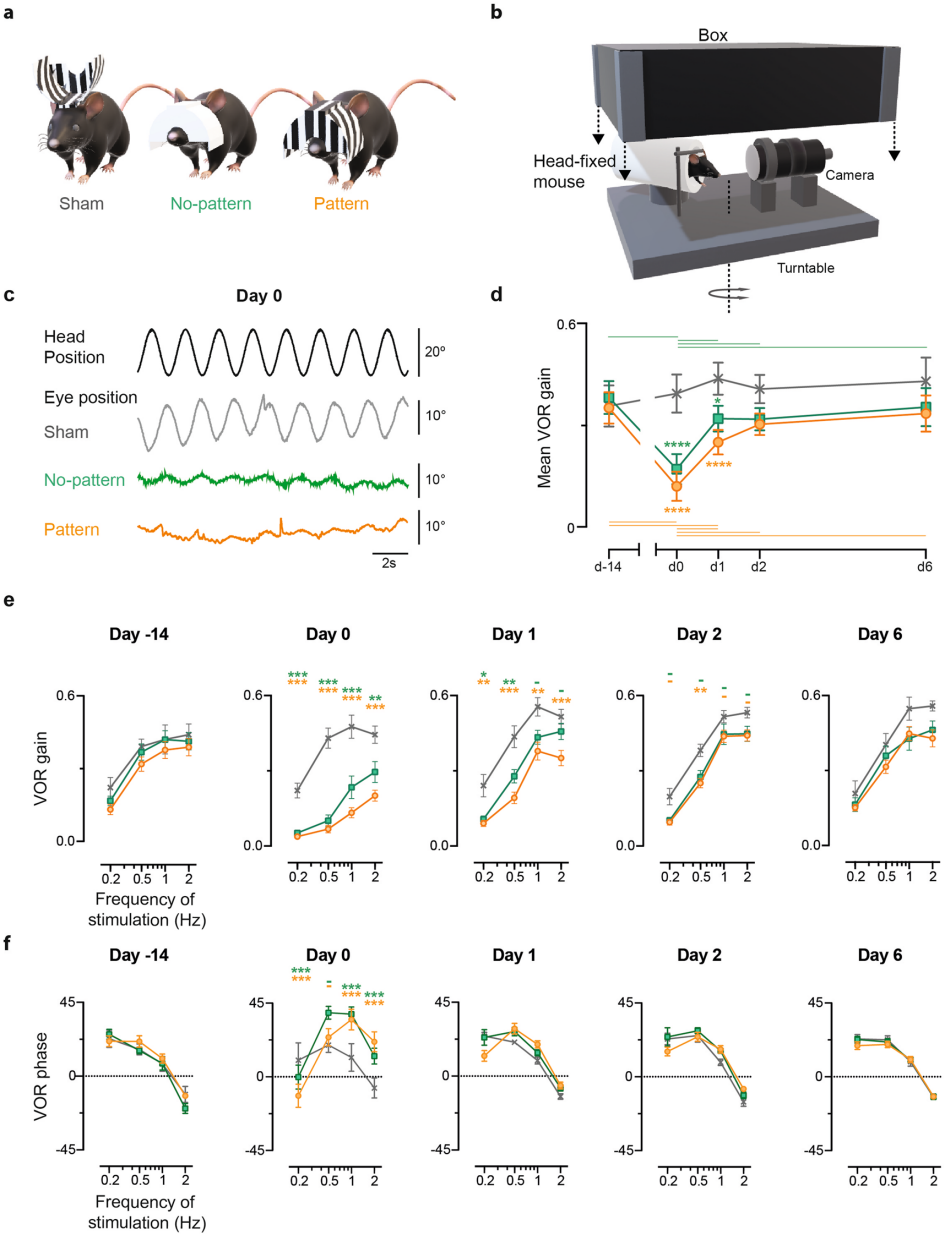
Effects of the VVM on the VOR. (**a**) Representation of mice wearing the three different types of devices used in the protocol; Pattern (left), No-pattern (middle) and Sham (right). (**b**) Depiction of the set-up to test VOR; the mouse is head-fixed and secured in a plexiglas tube during horizontal rotations of the turntable at a fixed peak velocity (30º/s) and variable frequencies (0.2-2 Hz). 3D renderings (a and b) were obtained using Paint 3D (Microsoft Corporation). (**c**) Example raw traces of VOR responses recorded on day 0 at 0,5 Hz for Sham (grey), No-pattern (green) and Pattern (orange). Head rotations in the dark evoked compensatory eye movements in the opposite direction. VVM-exposed mice showed altered eye movements. (**d**) Sham (n = 8), No-pattern (n = 16) and Pattern (n = 15) mean VOR gain along the entire protocol (20 days). The mean VOR (**e**) gain and (**f**) phase are at all tested days are plotted for the different frequencies of stimulation. The significance of the gain and phase changes compared to Sham are indicated next to each point in the graph. Horizontal lines represent intra-group significant differences between the tested days. Error bars represent ± SEM; Newman Keuls post-hoc test *p < 0.05; **p < 0.01; ***p < 10^–3^.

To measure the effect of each device (Fig. 1a; see “Methods”) on the VOR response, video-oculography was performed in darkness using sinusoidal turntable rotations (Fig. 1b). Figure 1c shows examples of the oculomotor response of mice of each of the three groups the day the device was removed (day 0, after 14 days of visuo-vestibular mismatch), during a 0.5 Hz stimulation at a fixed peak velocity of 30°/s. A striking difference can be qualitatively observed between the amplitude of the eye movements of Sham mice (n = 8) compared to No-pattern (n = 16) and Pattern (n = 15) mice, with smaller compensatory eye oscillation for the latter. Figure 1d quantitatively illustrates the evolution over time of the mean gain of the VOR for the three groups, which results in a statistically significant interaction between the effect of the tested *Day* and of the *VVM group* (repeated measures ANOVA, *Day *×* VVM group interaction effect:* F_8,108_ = 5.33, *p* < 10^–4^). As expected, before the visuo-vestibular mismatch the gain of the reflex was the same for the three groups of mice, and the Sham group did not show significant modulations over the whole duration of the protocol. On the other hand, at day 0, Pattern and No-pattern groups showed a significant decrease of the VOR gain and both had significantly lower gains than Sham (Newman–Keuls post-hoc test: at day 0 Sham vs Pattern, *p* < 10^–4^; Sham vs No-pattern, *p* < 10^–4^). At day 1, the gain of both Pattern and No-pattern groups had significantly recovered with respect to day 0 (Newman–Keuls post-hoc test: day 0 vs day 1, No-pattern, *p* = 0.0002; Pattern, *p* = 0.0004), but still showed significant differences compared to Sham (Newman-Keuls post-hoc test: at day 1, Sham vs Pattern, *p* < 10^–4^; Sham vs No-pattern, *p* = 0.019). From day 2 on, there was no significant difference between the VOR gain of the three *VVM groups*.

To determine whether the effect of the Pattern and No-pattern protocols on the VOR is modulated by the frequency of the stimulation, the VOR gain (Fig. 1e) and phase (Fig. 1f) are illustrated as bode plots for each test day. Frequency specific effects were revealed by the significant statistical interactions between the effects of the *Day*, *VVM group* and *stimulation Frequency*, on both the gain and the phase of the VOR (repeated measures ANOVA, *Day x VVM group x stimulation Frequency interaction effect*, Gain: F_24,324_ = 1.92, *p* = 0.007; Phase: F_24,300_ = 5.05, *p* < 10^–5^). More precisely, at day 0 the VOR gain of the Pattern and No-pattern groups dropped significantly compared to Sham (Fig. 1e) for all tested frequencies (Newman–Keuls post-hoc test, at day0 Sham vs Pattern at 0.2 Hz, *p* = 0.0002; at 0.5 Hz, 1 Hz and 2 Hz, all *p* < 10^–4^; Sham vs No-Pattern at 0.2 Hz, *p* = 0.0005; 0.5 Hz and 1 Hz, *p* < 10^–4^; at 2 Hz, *p* = 0.007). On day 1 the VOR gain of the Pattern group remained significantly reduced (Newman–Keuls post-hoc test, at day1 Sham vs Pattern at 0.2 Hz, *p* = 0.0014; 0.5 Hz, *p* = 0.0003; 1 Hz, *p* = 0.002; 2 Hz, *p* = 0.0006) whilst for the No-pattern group only the lowest frequencies (0.2 and 0.5 Hz) remained significantly reduced (Newman–Keuls post-hoc test, at day1, Sham vs No-pattern at 0.2 Hz, *p* = 0.0177; at 0.5 Hz, *p* = 0.0026).

On day 2 only the Pattern group VOR gain at 0.5 Hz is still different from the Sham (Newman–Keuls post-hoc test, at day2 Sham vs Pattern at 0.5 Hz, *p* = 0.0049). At day 6 significant differences can no longer be detected between the three VVM groups. Regarding the phase, Fig. 1f shows that the VVM had an effect only at day 0 and this effect is opposite for the low and high frequencies: greater phase lag at 0.2 Hz (Newman–Keuls post-hoc test, at day0: Sham vs No-pattern at 0.2 Hz, *p* < 10^–4^; Sham vs Pattern at 0.2 Hz, *p* < 10^–4^) and greater phase leads at 1 and 2 Hz (Newman–Keuls post-hoc test, at day0: Sham vs No-pattern and Sham vs Pattern at: 1 Hz and 2 Hz, all *p* < 10^–4^).

Overall, these results suggest that the VOR responses of the Pattern and No-pattern groups recovered a regular timing (phase) faster than an adequate amplitude of response (gain). Moreover, there were only limited differences in the gain reductions and recovery dynamics of the VOR responses between the Pattern and No-pattern groups. These results suggest that the high-contrast visual input of the Pattern device did not substantially influence the processes triggered by the visuo-vestibular mismatch.

### Effect of the VVM and recovery during sinusoidal optokinetic stimulation

**Figure 2:**
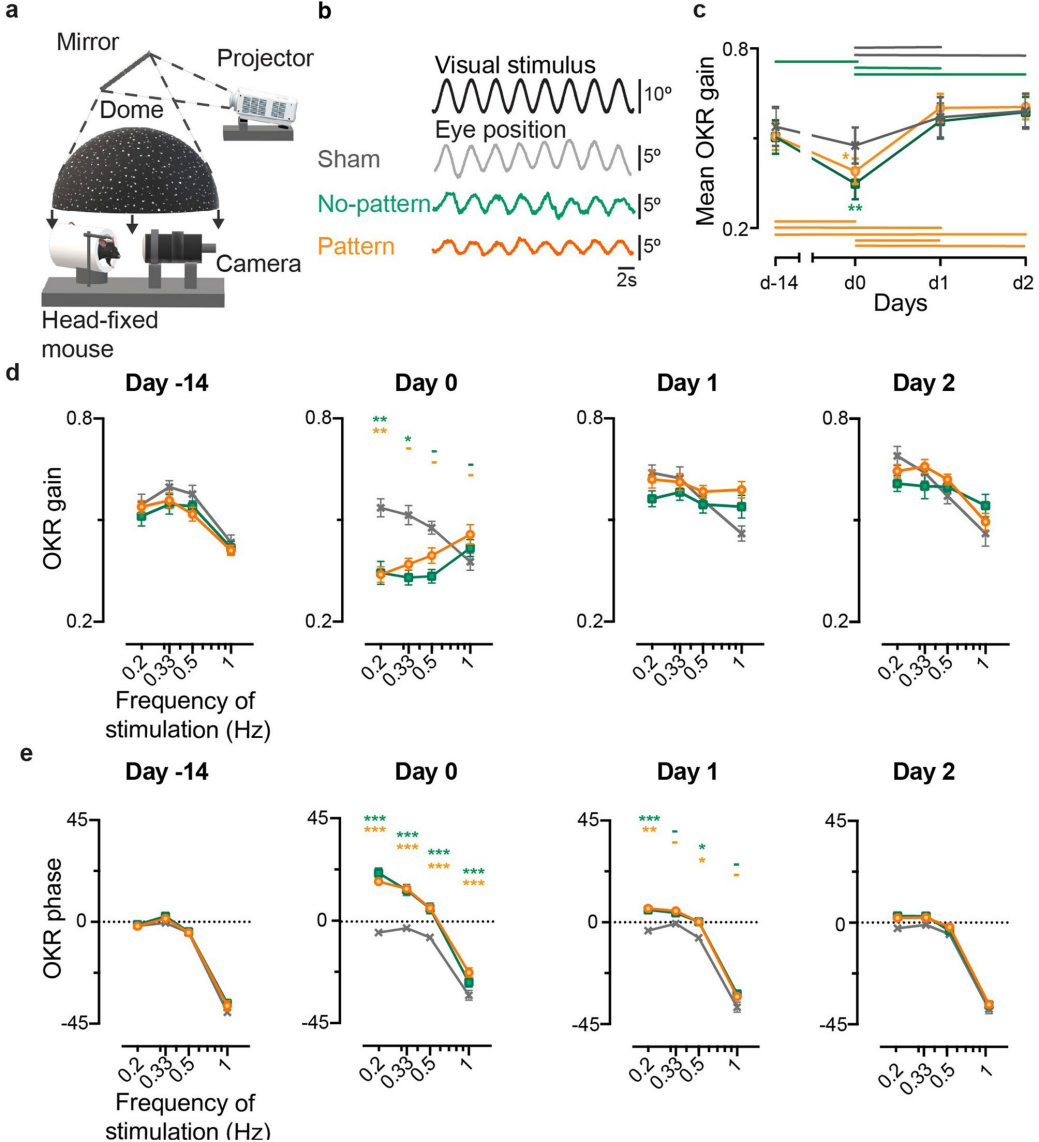
Effects of the VVM on OKR. (**a**) Representation of the set-up used to test OKR. A dotted background was projected into a mirror while the mouse was kept head-fixed. 3D model obtained using Paint 3D (Microsoft Corporation). (**b**) Example raw traces of the OKR response to a stimulation at 0.33 Hz at peak velocity of 10°/s for each condition; Sham (grey), No-pattern (green) and Pattern (orange). The eye position smoothly follows the optokinetic stimulus. (**c**) Kinematics of the mean OKR gain of Sham (n = 6), No-pattern (n = 8) and Pattern (n = 12) during the tested days. Horizontal lines represent intra-group significant differences between the tested days. OKR gain (**d**) and phase (**e**) at each of the different frequencies tested along each day. The significance of both gain and phase are indicated on top of each point in the graph for No-pattern (first line, green) and Pattern (second line, orange). Error bars represent ± SEM; Newman Keuls post-hoc test *p < 0.05; **p < 0.01; ***p < 10^–3^.

To investigate whether the visuo-vestibular mismatch affects the optokinetic reflex in the frequency domain, animals were first tested using sinusoidal rotations of a dotted pattern (Fig. 2a) at a fixed peak speed of 10°/s and at different frequencies in range 0.2–1 Hz. In response to sinusoidal oscillations of the dotted pattern, all mice responded with slow phases that smoothly tracked the visual stimulation (Fig. 2b). On day 0, a decrease in the amplitude of the eye movements was observed for No-pattern (n = 8) and Pattern (n = 12) groups, when compared to Sham (n = 6) (compare traces on Fig. 2b). Figure 2c illustrates for all VVM groups the global tendency for OKR gain changes along the tested days (repeated measures ANOVA, *Day x VVM group,* F_6,69_ = 2.2, *p* = 0.053). On day 0, the Pattern and No-pattern groups had a significantly diminished OKR gain (Newman-Keuls post-hoc test, at day0 Sham vs Pattern *p* = 0.0294; Sham vs No-pattern, *p* = 0.0046). On day 1, both Pattern and No-pattern groups showed a significant OKR gain recovery (Newman-Keuls post-hoc test, day0 vs day1: No-pattern, *p* = 0.0001; Pattern, *p* = 0.0002), resulting in no differences between the three groups from day 1 onwards.

To investigate if the alteration of the OKR response was dependent on the frequency tested, the gain (Fig. 2d) and phase (Fig. 2e) were plotted as a function of the stimulation frequency for each day. Frequency-specific effects were revealed by the significant statistical interactions between the effects of the *Day*, *VVM group* and *stimulation Frequency*, on both the gain and the phase of the OKR (repeated measures ANOVA, *Day x VVM group x stimulation Frequency interaction effect*, Gain: F_18,207_ = 2.6288, *p* = 0.0005; Phase: F_18,207_ = 3.2987, *p* < 10^–4^). Figure 2d shows that the above-mentioned effect at day 0 is mainly due to a reduction of the OKR gain for stimulations at the lowest tested frequencies (0.2 Hz and 0.33 Hz) (Newman-Keuls post-hoc test, at day 0 Sham vs Pattern at 0.2 Hz: *p* = 0.006; Sham vs No-pattern at 0.2 Hz: *p* = 0.0015; at 0.33 Hz: *p* = 0.0146). On this day, a significant phase shift towards a greater phase lead was also observed across all stimulation *Frequencies* for both Pattern and No-pattern groups (Newman–Keuls post-hoc test, day0, Sham vs Pattern at all frequencies: *p* < 10^–4^ ; Sham vs No-pattern at 0.2 Hz, 0.33 and 0.5 Hz: *p* < 10^–4^ ; at 1 Hz: *p* = 0.0078). On day 1, for all frequencies the OKR gain of both Pattern and No-pattern groups was no longer different from the Sham group. However, the timing of the OKR responses was still affected on Pattern and No-pattern groups, with remaining significant differences between these groups and Sham at 0.2 and 0.5 Hz (Newman–Keuls post-hoc test, day1, Sham vs Pattern at 0.2 Hz: *p* = 0.0001; at 0.5 Hz: *p* = 0.0296; Sham vs No-pattern at 0.2 Hz: *p* = 0.0004; at 0.5 Hz: *p* = 0.0282;). At day 2, both gain and phase had completely recovered, and no further differences were found between the three *groups*.

In conclusion, sinusoidal stimulations at different frequencies showed that significant gain and phase changes occurred in the OKR pathway following the VVM protocols. However, the reduction of the OKR responses appeared to last no more than one day and to predominantly affect the responses to low stimulation frequencies.

### Effect of the VVM in response to constant velocity optokinetic stimulation

**Figure 3:**
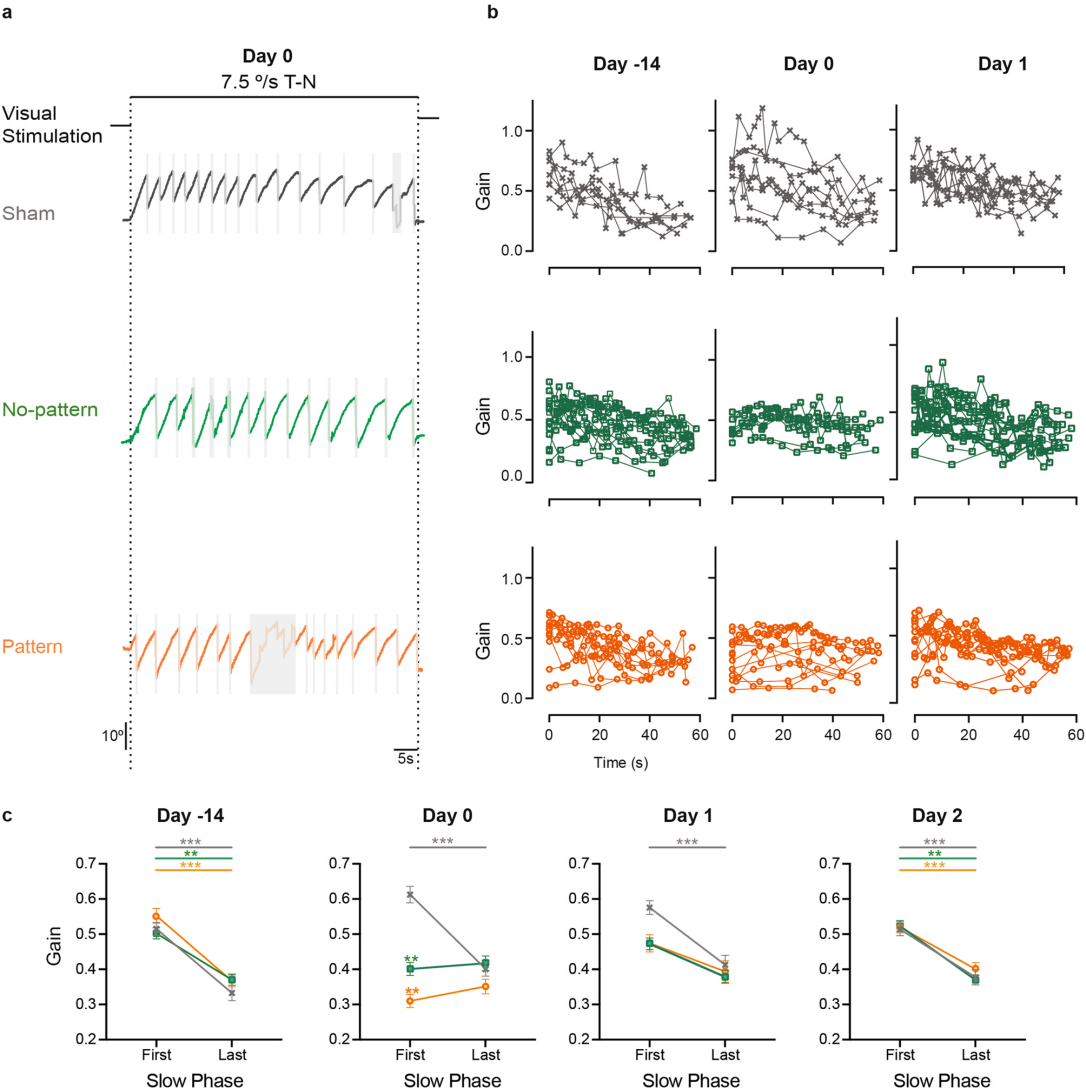
Effects of the VVM on OKR nystagmus. (**a**) Example raw traces for each group (Sham n = 8, grey; No-pattern n = 16, green; Pattern n = 15, orange) of the OKR response to the 60 s-long stimulation at 7.5º/s in temporo-nasal direction (black line). The grey rectangles mark the segments that were not considered in the analysis of the eye movements (quick phases or recording artefacts). Only slow phases were computed to characterize the response over the duration of the visual stimulation. (**b**) To illustrate the dynamic of responses over the stimulation period, the individual slow phases gains were plotted over time for 7.5°/s constant velocity stimulation in temporo-nasal direction. In the VVM-treated groups, the first response after stimulus onset (the first slow phase), appears lower after the VVM protocol. To quantify this difference, the gains of the first and last slow phases in response to all velocities and directions were averaged (**c**). When computed, the difference between the first and last SP gains reveals that before the VVM, the three groups had similar first and last gains while, on day 0, VVM-treated groups had lower first slow phase gains. On day 1, these gains have increased and at day 2, they are identical to Sham. The significant differences compared to Sham are indicated on top of each point in the graphs for No-pattern (green asterisks) and Pattern (orange asterisks) groups, respectively. Error bars represent ± SEM; Newman Keuls post-hoc test *p < 0.05; **p < 0.01; ***p < 10^–3^.

To further investigate how the optokinetic response is affected by the visuo-vestibular mismatch protocol, we also tested on another group of mice the OKR using long-lasting (60 s) full-field stimulations at constant velocities of 2.5, 5, 7.5 and 10°/s. Mice responded to the OKR stimulations with nystagmus-like responses consisting in series of slow phases in the direction of the stimulus, interrupted by quick-phases that recenter the eye in its orbit. Figure 3a displays example raw traces at day 0 at a velocity of 7.5°/s and Temporo-Nasal direction for Sham (n = 8), No-pattern (n = 16) and Pattern (n = 15) groups. Throughout each 60 s-long stimulation, the slow phases evoked were characterized by their number, amplitude and duration (at day 0, see Supplementary Fig. S1). For all groups, the number of slow phases significantly increased as a function of the stimulus velocity while their duration decreased (repeated measures ANOVA, *Velocity* effect, Number: F_3,90_ = 31.886, *p* < 10^–4^; Duration: F_3,93_ = 26.846, *p* < 10^–4^). No significant differences were found between the number and duration of slow phases between the three groups (repeated measures ANOVA, *VVM group* effect, Number: F_2,30_ = 1.3783, *p* = 0.27; Duration: F_2,31_ = 3.0007, *p* = 0.065). The amplitude of the slow phases also significantly increased with the stimulus velocity (repeated measures ANOVA, *Velocity* effect, Amplitude: F_3,93_ = 13.648, *p* < 10^–4^). However, the amplitude of the slow phases was significantly different between Sham and Pattern groups (repeated measures ANOVA, *VVM group* effect, Amplitude: F_2.31_ = 5.4568, *p* = 0.0093; Newman–Keuls post-hoc test, day0, Sham vs Pattern *p* = 0.0096).

The dynamics of the OKR response are illustrated on Fig. 3b; each line presents the gains of the slow phases performed, per mouse, along the stimulation at 7.5°/s at day-14, day0 and day1. As previously described in the mouse^26–28^, before the VVM protocol (day-14), the intensity of the optokinetic responses decreased over time during the 60 s duration of the optokinetic stimulation. This dynamic was quantified by comparing the mean gain of the first slow phase evoked after stimulus onset and the gain of the last slow phase evoked for all the different stimulations (2.5–10°/s) tested (Fig. 3c). There was a significant interaction between the *VVM Groups*, *Day* and *Component* (repeated measures ANOVA, *Day x VVM group x Component interaction effect*, Gain: F_6,90_ = 4.6741, *p* = 0.0035). Before the VVM (day-14), the mean gain of the first slow phases was significantly higher than the mean gain of the last slow phases for all the tested groups (Fig. 3c, day-14, Newman–Keuls post-hoc test, Sham, *p* = 0.0002; Pattern, *p* = 0.0002; No-pattern, *p* = 0.0013). At day 0, while this dynamic was unchanged in the Sham group, the response of Pattern and No-pattern groups showed rather different profiles. Specifically, the gain of the first slow phase was significantly reduced (day 0, Newman–Keuls post-hoc test; Sham vs Pattern, *p* = 0.0002; Sham vs No-pattern, *p* = 0.0008) while the gain of the last slow phase was not. At day 1, the mean gain of the first slow phases had mostly recovered for both Pattern and No-pattern groups (Newman–Keuls post-hoc test, day1, Sham vs Pattern, *p* = 0.3802; Sham vs No-pattern, *p* = 0.3399). However, the dynamic of their response was not yet back to normal (no significant decrease between first and last slow phase; Newman–Keuls post-hoc test, day1, First slow phase vs Last slow phase: Pattern, *p* = 0.0754; No-pattern, *p* = 0.0746). All these parameters had fully recovered on day 2.

Overall, the modifications of OKR responses observed during constant-velocity stimulation suggest that it was the transient, initial component of the OKR that was mostly affected by the visuo-vestibular mismatch, while the sustained, late component was not.

### Comparison between VOR and OKR gain changes as a result of the VVM protocol

**Figure 4:**
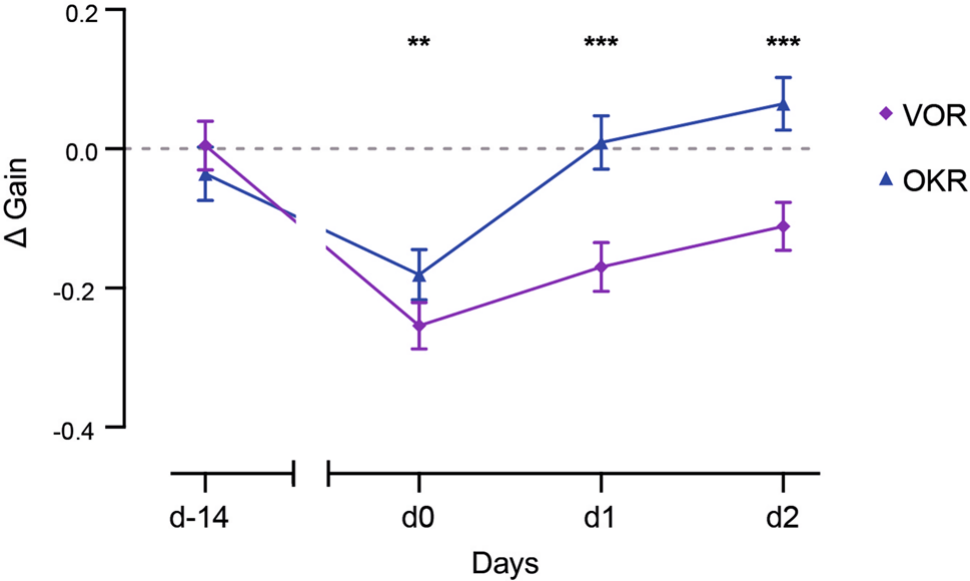
Comparison of the amplitude and dynamics of VOR and OKR gain changes over time. Global gain changes in VOR (purple diamonds) and OKR (blue triangles) over time were compared by subtracting the mean responses of the Sham group from those of the VVM-exposed mice (ΔGain). Differences between VOR and OKR responses of both VVM-exposed groups are represented with black asterisks. Error bars represent ± SEM; Newman Keuls post-hoc test *p < 0.05; **p < 0.01; ***p < 10^–3^.

Results presented so far demonstrate that 2 weeks of visuo-vestibular mismatch led to a significant alteration of the vestibulo-ocular reflex (Fig. 1) which recovered in about 2 days, while alteration of optokinetic reflex recovered in about 1 day (Fig. 2). Overall, the general effects were similar between Pattern and No-pattern mice. In order to directly compare the dynamics and magnitude of the changes in VOR and OKR while taking into account the non-specific effects, for each stimulation modality and day, we subtracted the mean responses of the Sham (VOR n = 8; OKR n = 6) group from those of the No-pattern (VOR n = 16; OKR n = 8) and Pattern (VOR n = 15; OKR n = 12) groups for all tested frequencies (ΔGain; Fig. 4). There was a significant interaction between the *Day*, and *Stimulation modality* (repeated measures ANOVA, *Day x Stimulation modality interaction effect*, F_3,117_ = 23.579, *p* < 10^–4^). At day-14, ΔGain for both VOR and OKR were, as expected, not different from 0, and not significantly different from each other (Newman–Keuls post-hoc test, d-14: VOR vs OKR, *p* = 0.1041). On day 0, day 1 and day 2, the ΔGain magnitude of VOR was significantly larger than the ΔGain for OKR at all tested days (Newman–Keuls post-hoc test, day0: VOR vs OKR *p* = 0.0014; day1 and day2: VOR vs OKR, *p* < 10^–4^). The ΔGain of OKR was only significantly smaller than the initial d-14 value at day0 (Newman–Keuls post-hoc test, day-14 vs day0 for OKR: *p* = 0.0001); the optokinetic responses were therefore no longer affected after the first day of recovery. On the other hand, the ΔGain of VOR was significantly different from day-14 at day 0 (Newman–Keuls post-hoc test, day-14 vs day0 for VOR, *p* = 0.0001), day 1 (day-14 vs day1 for VOR, *p* = 0.0001) as well as at day 2 (day-14 vs day2, *p* = 0.0001). This shows that, across the tested frequencies, VOR responses of Pattern and No-pattern mice are still abnormally reduced at day 2. Overall, this comparative analysis demonstrates that alteration in the OKR and VOR pathways differ both in terms of amplitude and dynamic of recovery.

## Discussion

This study shows a concurrent decrease of VOR and OKR following 14 days of a visuo-vestibular mismatch protocol. This concomitant reduction of OKR and VOR efficacy differs from what was previously reported following short–term (< 3 days) adaptation protocols in studies that also used mice as animal models^9,29^. These short-term VOR gain-down and gain-up adaptation protocols (1/2 h learning, up to 1 h follow-up) showed a moderate but significant increase in the OKR, in line with early reports in the rabbit^30^. It was suggested that, like during optokinetic learning, retinal slip might serve as a learning signal during short-term VOR adaptation^22,31^ and lead to potentiation in the optokinetic pathway^9^. Here, we used 2 different devices: one whose surface was completely blank (No-pattern), that would not generate any retinal slip when the eyes move, and another one with a high-contrast (Pattern). Our results show that both devices led to a significant reduction in both OKR and VOR, and no major alterations could be specifically ascribed to one or the other. The Pattern group showed larger VOR gain reductions as well as a slightly longer recovery time, but these differences were not compelling. From these experiments, it is therefore not possible to conclude whether the presence of the Pattern effectively led to a stronger “retinal slip”, which would represent an important error-signal driving the adaptation described in short-term protocols^31^. Another possibility would be that the retinal slip generated in the pattern group, and the complete absence of visual sensory feedback in the no-pattern group, would lead to comparable changes in VOR and OKR through completely different mechanisms. Moreover, the calibration of the VOR and OKR not only depends on retinal slip per se, but on the precise matching of different neural signals (e.g., head/eye velocities; relative phase of head vs eye movement^23^), it is therefore possible that the persistent perturbation of other gaze-related signals would be the main determining factor driving the changes observed in both groups.

Since the early 80s, long-term visual impairment protocols have been used in mammals to study the interaction of the vestibular and visual systems for gaze stabilization. The species used in these experiments vary from humans^32^ and non-human primates^33,34^ to cats^35,36^, rabbits^37,38^, and more recently, mice^20,21^. The inspiration for our protocol lies in the long-term adaptation experiments conducted in primates, in which the use of telescopic spectacles changed the visuo-vestibular interactions during head-free movements^33,34^. Several features of our results resemble the ones on these preceding studies. First, following a fixed-field protocol (fixed images viewed through lenses, providing a field of view fixed with respect to the head), the VOR in dark was decreased by 60–70% in 3–4 days^33^. The amplitude of this decrease is comparable to the decrease of VOR reported here following the VVM. OKR was also found to decrease by up to 50%, depending on the OKR velocity tested^39^. Specifically, the OKR deficit was positively correlated with velocities and concerned the closed-loop, velocity storage-dependent, pathway, as well as the optokinetic after nystagmus (OKAN) (see below^39^). Notably, comparable results were obtained during experiments in which animals were dark-reared to understand the role of visual inputs in the development of VOR and/or OKR. In rabbits that were dark-reared for 7 months^37^, the gain of the VOR in dark decreased ~ 70% at low frequencies and ~ 50% at high frequencies. The OKR gain also decreased by ~ 30%. OKAN responses were not affected. After 24 h in a normal light, both VOR and OKR increased and kept on improving for the next 3 months but remained permanently suboptimal^37^. In cats dark-reared until adulthood, the VOR in the dark was also found to be decreased by ~ 70%^35^. Again, the OKR was decreased by ~ 25% at low velocities, and even worsened as the velocity of the optokinetic stimulation increased. The velocity-storage dependent responses, including OKAN, were also markedly affected. It is noteworthy that, despite obvious visual and oculomotor differences, the abovementioned animal models confronted with protocols of long-term visual impairments, showed drastic reductions in VOR paralleled by a mild reduction of OKR efficacy. This observation would suggest that the basic mechanisms/circuitries involved are likely shared among mammals. Thus, the mouse model as well as VVM protocol can serve as valuable means to understand the signal-driven calibration of gaze stabilizing pathways.

While the VOR pathway constitutes a 3-neuron direct reflex arc^21^, several subcircuits contribute to different features of the optokinetic responses. Two distinct pathways related to different retinal ganglion cells appear, for instance, to manage the initial (transient) and late (sustained) phases of the OKR response^27^. In the following paragraph we discuss the results obtained using the transient sinusoidal and the constant-velocity lasting OKR tests, and their relations to VOR. In response to transient sinusoidal stimulation, we observed a frequency-selective decrease in the OKR responses. This decrease concerned the lowest frequencies tested (below 0.5 Hz). These results mirror the frequency-selective decrease observed in the VOR: at day 0, the larger gain reduction is observed for the lower frequencies tested (0.2 and 0.5 Hz). The VOR phase shift also differs between low (increased phase leads) and high (increased phase lags) frequencies. In the present freely behaving protocol, the visuo-vestibular mismatch mainly occurs in the range of natural head-movements. Previous studies have demonstrated that the mouse head movements produced during home-cage recordings or open-field active exploration are dominated by low frequencies^1,2,21^. The strongest effects observed on OKR and VOR at low frequencies could therefore relate to the statistics of the natural head-movements. Another explanation would be that the VVM mainly alters VOR/OKR in the range where visual information is the dominant sensory input signaling if eye movements are compensatory. Faulstich and colleagues^9^ demonstrated that, in mice, OKR and VOR have a mutually complementary working range; the OKR accounts for most of the gaze stabilization at frequencies below 0.5 Hz, while the VOR takes over above 0.5 Hz (mouse^9^, see also rabbit^30^). How the frequency-specific changes in OKR and VOR following VVM relates to the timing rules of plasticity in the flocculus^40,41^ and to the inter-dependency of these 2 reflexes^42^ should be the focus of future dedicated studies. Interestingly, other features of optokinetic response were revealed by the constant-velocity OKR tests. In particular, the gain of the first slow phase measured after stimulus onset was smaller in Pattern and No-Pattern mice compared to Sham (Fig. 3c). As the stimulation continued, the Sham mice OKR response decreased steadily (as previously reported^26–28^) whereas, Pattern and No-Pattern mice exhibited a consistently low response (Fig. 3b) that did not show any additional decrease. A degradation of the transient pathway could explain both the difference between the Pattern/No-Pattern and Sham mice during the sinusoidal stimulation and the loss in the initial OKR long-lasting response. In addition, a similar late OKR response for all mice groups suggests that the persistent pathway would be only marginally affected by the VVM protocol. Another mechanism involved in the response to a constant-velocity and lasting optokinetic stimulation is the velocity storage, which manifests in vertebrates as a build-up in the response during continued high velocity optokinetic stimulation and as a persistency in the response after stimulus offset^43,44^. However, in mice the velocity storage is considered “leaky”^45^ and does not generate much after nystagmus response (OKAN)^28,45^. A change affecting the velocity storage could therefore participate to the difference seen in the early, but not late, response to the long-lasting OKR responses. Since the velocity storage mechanism depends on the recruitment of vestibular brainstem circuitry^46–49^, whose excitability was shown to be decreased after VVM^42^, one hypothesis would be that the VVM impaired the capacity to recruit the brainstem circuitry common to OKR and VOR.

A key point in understanding the dynamic of plasticity of OKR and VOR networks, is the interaction between the cellular mechanisms located in the flocculi and the long-term consolidation of the motor learning in the brainstem^21, 50–52^. Jang and colleagues^53^ have recently demonstrated that the brainstem consolidation following a gain-up VOR training was dependent on the synergy of synaptic and intrinsic plasticity within Purkinje cells. In the case of an iterated VOR gain-up training, plastic brainstem modifications were reported as the combination of an increase in both the vestibular afferents-central vestibular neurons synapse as well as an increase in the excitability of central vestibular neurons. Notably, this result mirrors the results obtained by Carcaud and colleagues^21^ which demonstrated that a VOR gain reduction was accompanied by a decrease of the efficacy of the synapse between the vestibular afferents and the central vestibular neurons, accompanied by a decrease in the excitability of a subpopulation of central vestibular neurons. Collectively, these studies demonstrate that VOR gain modifications depend on synaptic and intrinsic plastic changes in the brainstem.

Overall, the visuo-vestibular mismatch protocol performed in freely behaving mice led to a frequency-specific reduction of the gain of both VOR and OKR. The changes observed in the OKR were of lower intensity and recovered more rapidly than the changes in the VOR. Based on the recent findings on cellular mechanisms associated with learning in gaze stabilization networks, future experiments conducted on mouse and model studies will help to better understand the cellular mechanisms associated with OKR/VOR tuning and their interactions.

## Methods

### Ethics

A total of 65 male C57/BL6J mice, age 6–10 weeks, was used for the described protocol. Animals were used in accordance with the European Communities Council Directive 2010/63/EU. All efforts were made to minimize suffering and reduce the number of animals included in the study. All procedures were approved by the ethical committee for animal research of the University of Paris (CEEA.34).

### Headpost implantation surgery and animal care

Surgical procedures, postoperative care, device fixation and animal surveillance during the protocol were performed as described previously^20,21^. Briefly, mice anesthetized with isoflurane gas had their heads shaved with small clippers. Then, lidocaine hydrochloride (2%; 2 mg/Kg) was injected locally before a longitudinal incision of 1.5 cm was made to expose the skull. Just anterior to the lambda landmark, a small custom-built headpost (3 × 3 × 5mm; poly lactic acid) was cemented (C&B Metabond; Parkell Inc., Edgewood, NY) and laterally covered with resin (Heraeus) for protection. Animals were fully recovered 30 min after the end of the surgery, yet buprenorphine (0.05 mg/kg) was provided for postoperative analgesia and they were closely monitored for the following 48 h.

### Long-term visuo-vestibular mismatch protocol

The custom-built devices were secured on top of the mouse headpost for 14 days. The device consisted of a 0.9 g helmet-like structure that completely covered the mouse's head. The front of the device was adapted to the animal’s anatomy so that the nose was not covered, and its width allowed for grooming and barbering behaviors. Given its dimensions^20^ and small distance to the eye (5 mm), the device completely covers the entire binocular field and monocular horizontal fields of view^54^, and most (85%) of the field on the vertical axis. The lower edges of the device are in the part of the visual field that the mouse would normally cover with head movements^55,56^ and which is poorly represented in retinotopic space^57^. To preserve light-dependent physiology and nychthemeral rhythm, the device was made of slightly opaque PLA so that the animal could no longer see the surrounding, but still received luminance stimulation. Design and 3D printer specifications of this device were previously detailed^20^. VOR and OKR responses were recorded before the beginning of the VVM protocol. Then, mice were designated to 1 of the 3 visuo-vestibular mismatch devices (Fig. 1a): Sham (n = 14), ‘No-pattern’ (n = 24) or ‘Pattern’ (n = 27). ‘No-pattern’ devices were blank (white PLA color) while ‘Pattern’ ones had black 3 mm stripes drawn onto their surfaces. These stripes were previously used to create a high-contrast fixed visual signal during self-generated head movements^21^. Sham mice had the device fixed upside-down so that they were exposed to the same procedure except for the visuo-vestibular mismatch^21^. After securing the device into the headpost with a pair of screws, it was ensured that the nose of the mouse was aligned with the center of the device’s snout aperture and that no pressure was being directly applied to it.

Animals were housed in groups of 5 composed of at least 1 mouse of each condition. Moistened food and hydrogel were placed on the cage’s floor to facilitate feeding on the first two days after the fixation of the device. Mice were left with the implanted devices for 14 days. To ensure their well-being, mice were daily weighted and surveilled. After this two-week period, the device was removed and video-oculography was performed at different timepoints.

### Video-oculography recording sessions

Eye movements were recorded with a non-invasive video-oculography system (ETL-200, Iscan; acquisition rate 120 Hz) following the methodology previously described (Stahl et al. 2000). Eye and Image/head position signals were sampled at 1 kHz, digitally recorded (CED power1401 MkII) with Spike 2 software and analyzed off-line in Matlab (Matlab, The MathWorks, Natick, MA, USA; RRID: SCR:001622) programming environment. The experimental set-up (see Figs. 1b and 2a) and methods of data acquisition are akin to those described in^21,58^. In sum, mice were put in a custom-built Plexiglas tube and head-fixed at ~ 30° nose-down position to align the horizontal canals to the yaw plane^59,60^. This restraint assembly was fixed on a rotating platform on top of an extended rig with a servo-controlled motor. Recording sessions were performed in a temperature-controlled room (21 °C) and lasted up to 45 min. To study the effects of the device on the gaze stabilizing reflexes, mice underwent a recording session before (*day-14*) having the device implanted, immediately after its removal (*day0*), 1 (*day1*), 2 (*day2*) and 6 (*day6*) days afterwards. To minimize the exposure to the visual scene, the recording session on *day 0* began immediately after the device was removed in a room with low luminance. Mice were then left in standard lighting and housing conditions.

### Vestibulo-ocular reflex tests and analysis

VOR tests were performed with all sources of light turned off except for computer screens. The turntable was surrounded by a black box to isolate the animal from any remaining light, creating a final luminance inside the box of < 0.02 lx (Luxmeter Lux-1337 Iso-tech). To prevent excessive pupil dilatation, pilocarpine 2% was instilled into the eye.

To evaluate the angular vestibulo-ocular reflex, different vestibular stimulations were used. Horizontal vestibulo-ocular reflex (VOR) recordings consisted of sinusoidal angular rotations around the vertical axis at 0.2, 0.5, 1 and 2 Hz at a peak velocity of 30˚/s. The angular amplitude of the table movement was adjusted accordingly. At least 10 cycles were analyzed *per* frequency/velocity. The compensatory eye movements were studied by calculating their gain and phase in each condition. Briefly, the gain is the ratio between the response (eye) velocity and stimulus (head) velocity. The phase is the temporal shift between the eye and table rotations, expressed in degrees of the sinusoidal cycle. Detailed VOR gain and phase calculation are reported in^21^.

### Optokinetic reflex tests and analysis

To record the OKR, head-fixed mice were surrounded by a 40 cm wide dome (see Fig. 2a) and all sources of light were turned off except for the optokinetic stimulus projector. Optokinetic stimulations consisted in 25,000 white dots (max width 0.075°) randomly distributed on a black background. The light intensity inside the dome during the test was around 185 lx.

Two types of optokinetic stimuli were tested: *sinusoidal* and *constant velocity* stimulations. The *sinusoidal* one allowed a direct comparison with the VOR responses in the frequency domain^9^, whilst the classical *constant velocity* stimulation allowed better discriminating between the transient and sustained responses of the OKR^27^. To avoid cross-effects, both the two OKR tests were performed on different groups of mice.

The *optokinetic sinusoidal stimulations* were tested *at different frequencies (0.2; 0.33, 0.5; 1 Hz; peak velocity of 10°/s)* using monocular stimulation*.* OKR response was expressed as gain and phase determined by least-squares optimization method, similar to the VOR gain and phase^21^.

*Optokinetic constant velocity (2.5; 5; 7.5; 10°/s) binocular stimulations in alternated clockwise (or naso-temporal (N-T) to the recorded eye) and counterclockwise (or temporo-nasal (T-N) to the recorded eye) directions*. On each day, mice were tested during a unique trial in each direction and each velocity, that is a total of 8 different trials/day. The stimulation lasted 60 s and was separated by at least 60 s of darkness. Analysis of the generated optokinetic nystagmus (alternation of slow and quick phases^61^) was performed on the slow phases after automatic removal of the quick phases using a detection threshold set at 50°/s. Slow phases shorter than 1 s were discarded. To study the individual dynamics of slow phases, each was fitted to an exponential curve in order to reduce noise in parameter estimation. The slow phase gain was computed as the ratio between the amplitude of the interpolated eye displacement and the amplitude of the stimulus displacement, on the same time window. The ocular response was analyzed for the entire duration of the stimulation and the gain of the initial and last slow phase was used to describe its transient and persistent components respectively. The mean number, amplitude and duration of the slow phases during the stimulation were also computed.

### Statistical analyses

For both VOR and OKR sinusoidal stimulation experiments the effect of the protocol on the reflex gain and phase were statistically tested by performing a mixed-model ANOVA with the *VVM group* (Sham, Pattern and No-pattern) as between-individual independent factor and the Day (d-14, day0, day1, day2, and, for VOR only, day6) and the stimulation *Frequency* (for VOR: 0.2, 0.5, 1, 2 Hz; for OKR 0.2, 0.33, 0.5, 1 Hz) as within-individual independent factors. The main effects of these factors, as well as their interactions, were tested.

For the continuous OKR stimulation a mixed-model ANOVA was used to test the effect and the interaction between the following independent variables: *VVM group* (Sham, Pattern and No-pattern), as between-individual factor, and the *Day* (d-14, day0, day1, day2), stimulation *Velocity* (2.5, 5, 7.5, 10°/s), *Direction* (T-N, N-T) and *Component* [transient (first slow phase) and sustained (last slow phase)] as within-individual independent factors. A mixed-model ANOVA was also used for evaluating the effects of the *VVM group, Velocity* and *Direction* on the number, amplitude and duration of the OKR slow phases at day0.

For the comparison between the OKR and VOR sinusoidal stimulation experiments, for each stimulation modality and for each day, we first subtracted the average gain of the Sham group to those of the Pattern and No-pattern group. Then, we applied a mixed-model ANOVA with the *stimulation modality* (OKR and VOR) as between-individual independent factors and the *Day* (d-14, day0, day1, day2) as within-individual independent factors. For this analysis the mice of Pattern and No-pattern groups were pooled together.

For all analyses the significance threshold was set at *p* < 0.05 and Newman–Keuls post-hoc test was performed whenever a significant main effect or interaction was detected. Data were analyzed using Statistica (StatSoft Inc.) software.

## Supplementary information

Supplementary Informations.

## Data Availability

The VVM protocol used in this study is explained in detail in the published article^20^. The datasets generated during the current study are available in the Mendeley Data repository, https://dx.doi.org/10.17632/r56jxvyycv.1.
