# Supplementary material for: Long term visuo-vestibular mismatch in freely behaving mice differentially affects gaze stabilizing reflexes
Source: Sci Rep. 2020 Nov 18;10:20018. doi: 10.1038/s41598-020-77026-w (PMC7674424; doi:10.1038/s41598-020-77026-w)
Supplement: Supplementary file 1 — Supplementary Informations. [file 41598_2020_77026_MOESM1_ESM.pdf]

Title: **Long term visuo-vestibular mismatch in freely behaving mice differentially affects gaze stabilizing reflexes**

Authors: Filipa França de Barros[1]*, Louise Schenberg[1], Michele Tagliabue[1], Mathieu Beraneck[1]*

Affiliation: [1]Université de Paris, CNRS, Integrative Neuroscience and Cognition Center, F-75006 Paris, France.

*Corresponding authors:
Filipa França de Barros: CNRS UMR 8002, Université de Paris, 45 rue des St-Pères, Paris 75270, France. E-mail: filipa.barros@parisdescartes.fr

Dr. M. Beraneck: CNRS UMR 8002, Université de Paris, 45 rue des St-Pères, Paris 75270, France. E-mail: mathieu.beraneck@parisdescartes.fr

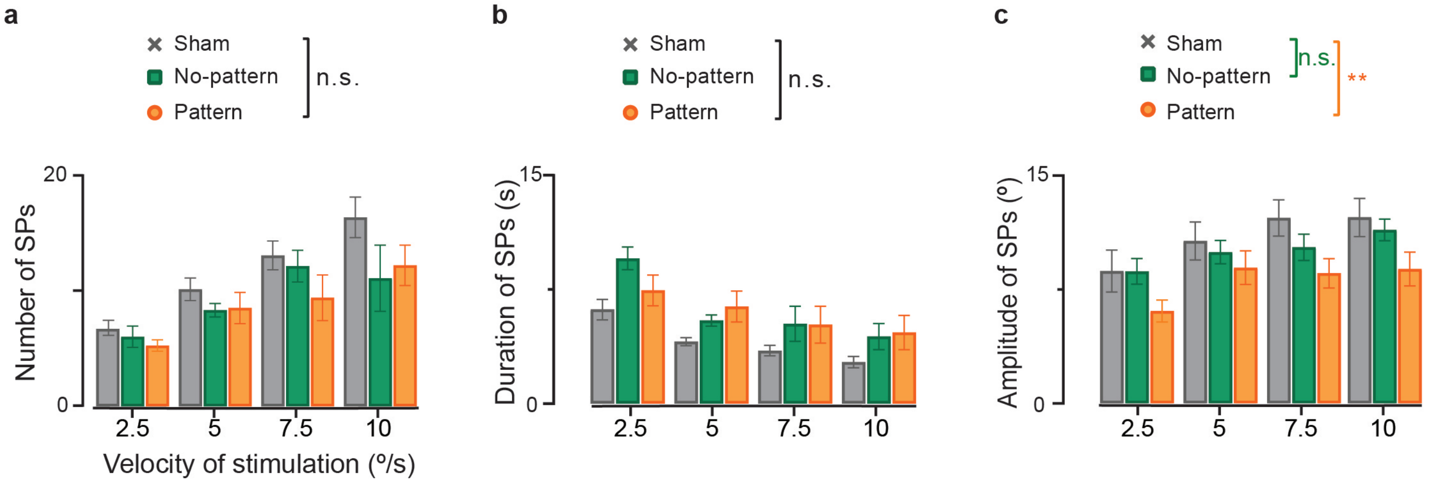

Supplementary Figure S1. Features of the optokinetic nystagmus. Quantification of all the slow phases evoked along the constant velocity 60s-long optokinetic stimulation. The (a) number, (b) duration and (c) amplitude describe the slow phases recorded at day 0 at different velocities. Differences between groups are indicated above each graph. Bars represent mean+/-SEM. Newman Keuls post-hoc test * p<0.05; **p<0.01; ***p<10-3.
